# Endometrial regeneration with mesenchymal stem cells and exosomes: an experimental rat model of intrauterine adhesions

**DOI:** 10.1038/s41598-026-45939-7

**Published:** 2026-03-26

**Authors:** Feride Atay, Arif Onur Atay, Ali Akdemir, Yigit Uyanikgil, Ebru Eroglu, Gulinnaz Ercan, Rozita Tamjidifar

**Affiliations:** 1 Department of Obstetrics and Gynecology, Ege University Faculty of Medicine, Izmir, Turkey; 2 Department of Obstetrics and Gynecology, Torbali State Hospital, Izmir, Turkey; 3 Department of Histology and Embryology, Ege University Faculty of Medicine, Izmir, Turkey; 4 Department of Medical Biochemistry, Ege University Faculty of Medicine, Izmir, Turkey

**Keywords:** Cell biology, Diseases, Medical research, Stem cells

## Abstract

Intrauterine adhesions (IUA) arise from inflammation-driven fibrotic remodeling that limits endometrial repair. Mesenchymal stem cells (MSCs) and MSC-derived exosomes are promising, but direct comparisons by tissue source and delivery route are limited. To compare umbilical cord (UC) and adipose-derived MSCs versus their exosomes and to assess local intrauterine versus intravenous (IV) UC-exosome delivery in a rat IUA model. Thirty-five female Sprague–Dawley rats were randomized (*n* = 5/group) to negative control, positive control (ethanol-IUA), and five treatment arms (local UC-MSC, UC-Exo, Adipose-MSC, Adipose-Exo, and IV UC-Exo). IUA was induced with 0.3 mL 95% ethanol; treatments were administered 2 weeks later. Two weeks after treatment, uterine tissues underwent H&E/Masson’s trichrome evaluation, epithelial and wall thickness measurements, and blinded 0–3 scoring of inflammation, vascular proliferation, and fibrosis. Positive controls showed severe fibrosis and luminal narrowing. All treated groups demonstrated improved architecture and reduced collagen deposition versus positive controls. Epithelial thickness was higher in treated groups but did not differentiate treatment arms; only negative vs. positive controls differed (*p* < 0.001). Uterine wall thickness increased in all treatment groups versus positive controls (overall *p* < 0.006), and local and IV UC-Exo groups were similar. Remodeling scores (inflammation/fibrosis) separated groups more clearly, with the most favorable combined profile in the Adipose-Exo group. MSC-based interventions (especially exosomes) attenuated inflammatory–fibrotic remodeling in this rat IUA model. Tissue source and product type influenced the repair profile while IV exosome delivery produced remodeling effects comparable to local administration.

**Supplementary Information:**

The online version contains supplementary material available at 10.1038/s41598-026-45939-7.

## Introduction

Intrauterine adhesions (IUA), also known as Asherman syndrome, typically develop following trauma or infection that damages the basal layer of the endometrium, resulting in fibrotic bands bridging opposing uterine walls and leading to partial or complete obliteration of the uterine cavity and/or cervical canal^1,2^. Clinically, IUA is classically associated with menstrual disturbances such as hypomenorrhea or amenorrhea, infertility or subfertility, and recurrent pregnancy loss^1,2^. Importantly, accumulating clinical evidence indicates that even after adhesions are identified and treated, reproductive outcomes may remain compromised, including reduced live birth rates and prolonged time to live birth, suggesting persistent endometrial dysfunction and highlighting the challenge of achieving durable functional endometrial restoration^3,4^.

Hysteroscopic adhesiolysis is widely recognized as the primary treatment for intrauterine adhesions (IUA; Asherman syndrome) and is commonly supplemented with postoperative adjuvant measures aimed at preventing re-adhesion. These strategies include estrogen therapy to promote endometrial regeneration, as well as mechanical separation using intrauterine balloon catheters or intrauterine devices^2,5^. Despite such interventions, clinically significant recurrence rates remain common, particularly in moderate-to-severe disease, with reported rates of adhesion reformation after hysteroscopic surgery reaching up to 28.7%^6^. In addition, pre-existing inflammatory conditions, such as chronic endometritis, may exacerbate fibrotic remodeling and further compromise reproductive outcomes^4^. Notably, even following apparently successful adhesiolysis, long-term reproductive performance often remains suboptimal, suggesting incomplete functional recovery of the endometrium^3^. Collectively, these observations indicate that current therapeutic approaches primarily address mechanical restoration of the uterine cavity rather than true biological regeneration of endometrial tissue, underscoring the need for advanced regenerative strategies^7^.

Mesenchymal stem cells (MSCs) have demonstrated regenerative effects in experimental models of intrauterine adhesions (IUA) and endometrial injury, with studies showing improvements in histological repair and functional recovery in Asherman syndrome models, including restoration of endometrial architecture and receptivity^1,8,9^. Increasing evidence indicates that these therapeutic benefits are mediated predominantly through paracrine mechanisms rather than durable cellular engraftment or direct differentiation, consistent with broader findings in regenerative medicine emphasizing the role of the MSC secretome^10^. Within this paracrine framework, MSC-derived exosomes and extracellular vesicles have emerged as key effectors capable of modulating fibrosis, angiogenesis, inflammation, and tissue repair in models of endometrial damage and IUA^1,9,10^. Importantly, exosome-based therapies represent a cell-free regenerative strategy that may offer practical translational advantages over cell-based approaches, including improved safety, reduced immunogenicity, and greater potential for standardization and storage^10,11^.

Although mesenchymal stem cell (MSC)– and extracellular vesicle (EV)/exosome–based strategies are increasingly explored for endometrial repair, existing studies on intrauterine adhesions (IUA) typically evaluate a single MSC tissue source within each experimental framework (for example, adipose-derived MSC exosomes in preclinical models versus umbilical cord–derived MSCs in early translational settings) resulting in limited direct comparative evidence across tissue sources, such as adipose versus umbilical cord origin^1,12^. Similarly, most reports in IUA and endometrial injury models investigate either cell-based MSC therapy or MSC-derived EV/exosome therapy in isolation, leaving head-to-head comparisons between cellular and cell-free approaches largely unexplored^12,13^. In addition, the route of administration may critically influence therapeutic efficacy, as effective uterine homing and tissue retention remain challenging following systemic delivery; however, systematic comparisons of local intrauterine versus intravenous administration in IUA models are scarce^12,13^. Accordingly, the present study was designed to comparatively evaluate MSCs and MSC-derived exosomes from different tissue sources and to assess the impact of delivery route on histological and regenerative outcomes in a rat model of intrauterine adhesions.

## Materials and methods

### Study approvals

This study received academic board approval from the Department of Obstetrics and Gynecology, Ege University Faculty of Medicine Hospital (25 January 2021; No: E.26126) and ethical approval from the Ege University Local Ethics Committee for Animal Experiments (Approval No: 2021-036; 28 April 2021). The study was supported by the Scientific Research Projects Coordination Unit (BAP) of Ege University (Project No: 23335). Following an amendment due to inability to procure cord blood–derived stem cells, an additional ethics approval was obtained to implement protocol modifications.

### Animals and experimental groups

The animals used in this study were obtained from the Ege University Laboratory Animals Application and Research Center (HAYMER), Izmir, Türkiye. A total of 35 adult female Sprague–Dawley rats (approximately 12 weeks old, ~ 200 g) were housed under standard laboratory conditions with ad libitum access to food and water. Animals were randomly assigned into seven groups (*n* = 5 per group):

1. *Negative control (N-C)*: no intervention.
2. *Positive control (P-C)*: intrauterine adhesion model induced with ethanol, no treatment.
3. *UC-MSC*: ethanol-induced model + local umbilical cord–derived MSCs.
4. *UC-Exo*: ethanol-induced model + local exosomes derived from umbilical cord MSCs.
5. *Adipose-MSC*: ethanol-induced model + local adipose-derived MSCs.
6. *Adipose-Exo*: ethanol-induced model + local exosomes derived from adipose MSCs.
7. *IV-UC-Exo*: ethanol-induced model + intravenous exosomes derived from umbilical cord MSCs.

### Induction of intrauterine adhesions

**Fig. 1:**
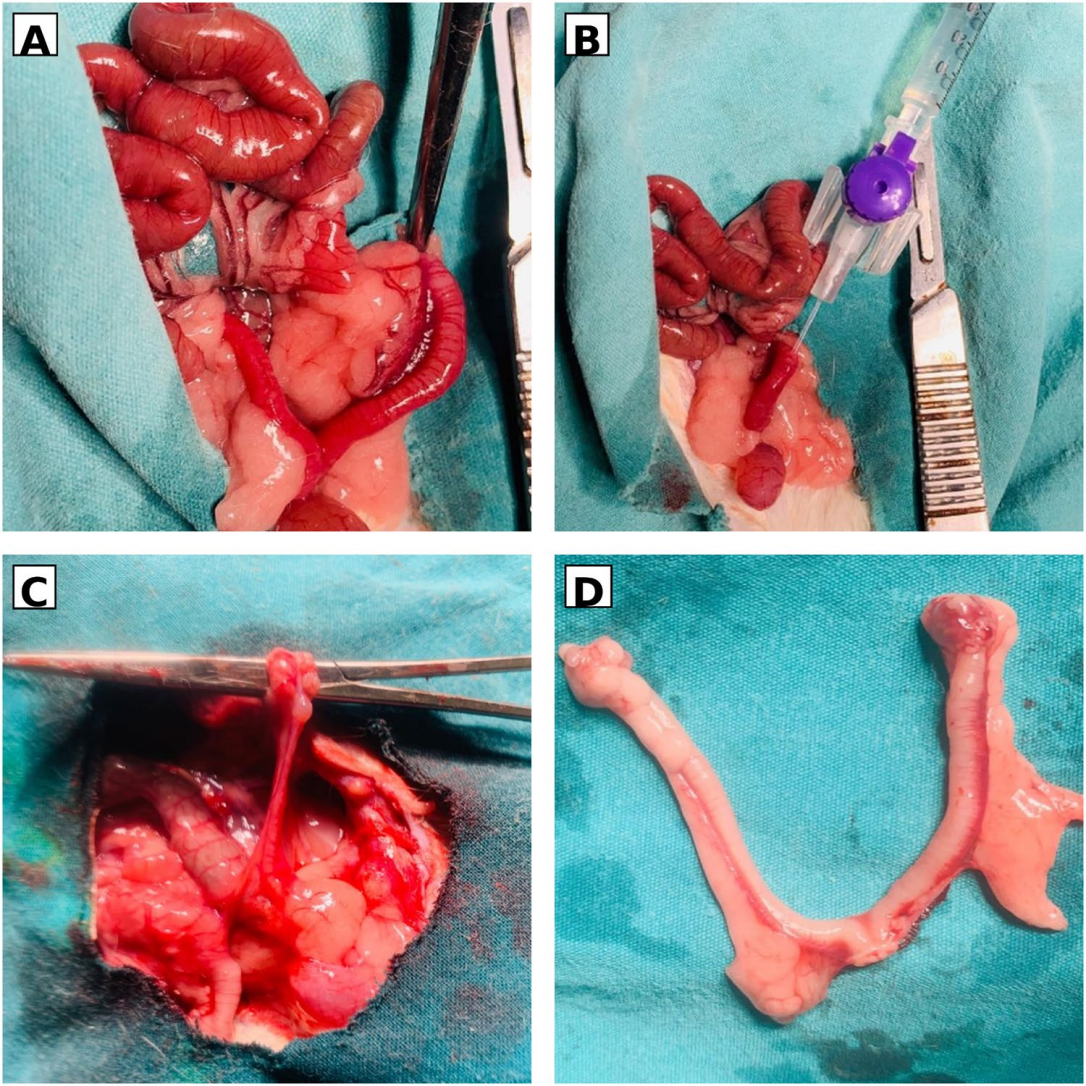
Experimental workflow for intrauterine adhesion induction and therapeutic administration in the rat model. (**A**) Midline laparotomy and abdominal exploration with exposure of the uterus. (**B**) Intrauterine cannulation and ethanol administration to induce chemical endometrial injury. (**C**) Representative macroscopic appearance of the uterine horn after ethanol-induced adhesion formation. (**D**) Representative hysterectomized uterus specimen collected at the end of the treatment period.

Intrauterine adhesions were induced using a chemically mediated injury model adapted from previously described protocols. Following intraperitoneal anesthesia with ketamine (50 mg/kg) and xylazine (5 mg/kg), a midline laparotomy was performed. The endometrial injury model was established by intrauterine injection of 95% ethanol, as previously described by Zhang et al.^14^. In one uterine horn, a 26G cannula was inserted into the intrauterine cavity near the tubal end, and 0.3 mL of 95% ethanol was slowly administered. After 3 min, the uterine cavity was irrigated with 0.5 mL of normal saline. The abdominal wall was closed anatomically, and a 2-week interval was allowed for adhesion formation. The experimental procedure is illustrated in Fig. 1.

### **Characterization of MSCs and MSC-derived exosomes**

Adipose tissue-derived and umbilical cord-derived MSCs were cultured under standard conditions and exhibited the typical adherent, spindle-shaped fibroblast-like morphology. Flow cytometric characterization demonstrated positive expression of CD90, CD73, and CD105, with CD44 evaluated as an additional supportive marker. PE-only staining was negligible, indicating low background signal. MSC-derived exosomes were characterized by scanning electron microscopy (SEM) and by flow cytometric assessment of the exosome-associated markers CD9 and CD63. Detailed characterization procedures are provided in the Supplementary Methods.

### Therapeutic administration

Two weeks after adhesion induction, a second laparotomy was performed in the relevant treatment groups. Prepared MSC or exosome suspensions were administered locally into the intrauterine cavity in a standardized volume of 0.2 mL, according to group allocation. In the systemic treatment group, umbilical cord MSC–derived exosomes were administered intravenously via the tail vein. All surgically treated animals received intramuscular penicillin (1000 U/kg) for infection prophylaxis. A schematic overview of the experimental workflow is provided in Fig. 1. At the end of the experimental protocol, animals were euthanized by cervical dislocation (physical method), performed by trained personnel in accordance with the approved institutional animal ethics protocol. No chemical euthanasia agent was used.

### Histological and Immunohistochemical Evaluation

Two weeks after treatment administration, animals were euthanized and uterine tissues were harvested for analysis. Sections were stained with hematoxylin–eosin and Masson’s trichrome for histological evaluation. Immunohistochemistry was performed for FGF-2, VEGF, HIF-1α, and collagen type I. Semi-quantitative scoring was conducted by blinded observers. For each animal, histological and immunohistochemical analyses were performed on one uterine horn. Detailed staining protocols and scoring criteria are described in the Supplementary Methods.

### Microscopic evaluation and semi-quantitative scoring

Microscopic evaluation was performed using a light microscope (Olympus BX51) equipped with an integrated digital camera (Olympus C-5050). Immunohistochemical staining was assessed in five randomly selected ×40 high-power fields per specimen, and staining distribution/intensity was scored on a 0–3 scale. Inflammation, vascular proliferation, and fibrosis were evaluated using predefined semi-quantitative criteria on a 0–3 scale, based on the examination of 10 randomly selected fields per specimen, in accordance with previously published experimental rat studies^15,16^. All histological and immunohistochemical assessments were independently performed by two histologists blinded to group allocation. Detailed scoring criteria are provided in Appendix A (Supplementary Methods).

### Statistical analysis

Statistical analyses were performed using IBM SPSS Statistics for Windows, version 25.0 (IBM Corp., Armonk, NY, USA). Normality of numerical variables was assessed using the Shapiro–Wilk test based on residual values obtained after analysis of variance. Variables showing no significant deviation from normal distribution (histological score means) were compared among groups using one-way analysis of variance (ANOVA). Homogeneity of variances was evaluated with Levene’s test, and as no significant heterogeneity was detected, Tukey’s HSD test was used for post hoc pairwise comparisons. Numerical variables not conforming to normal distribution (thickness variables) were compared using the Kruskal–Wallis test, followed by Dunn’s test with Bonferroni correction for pairwise comparisons. In addition, histological scores assessed across 10 domains were compared among groups using the chi-square test, and z-scores for individual cells of the contingency tables were calculated to identify categories deviating from independence. All hypothesis tests were two-sided, and a p value of < 0.05 was considered statistically significant.

## Results

### MSC and exosome characterization results

Flow cytometric analysis confirmed the mesenchymal phenotype of both adipose tissue–derived and umbilical cord–derived MSCs. Adipose-derived MSCs showed high expression of CD90 (99.92%), CD105 (98.12%), CD44 (92.88%) and CD73 (97.06%) with negligible PE-only staining (0.08%), and umbilical cord–derived MSCs showed a similar phenotypic profile. Both MSC populations also exhibited the typical spindle-shaped, fibroblast-like, adherent morphology under phase-contrast microscopy. Exosomes derived from both MSC sources were characterized by SEM and flow cytometry and showed positivity for CD9 (71.18%) and CD63 (79.14%). These results confirmed successful characterization of the MSCs and their derived exosomes prior to in vivo administration.

### Endometrial epithelial thickness

Endometrial epithelial thickness was calculated for each animal as the mean of measurements obtained from five distinct microscopic fields. Group-level thickness values are summarized in Table 1. Compared with the positive control group, all treatment groups showed higher endometrial epithelial thickness; however, these increases did not reach statistical significance. A significant difference was observed only between the negative and positive control groups (*p* < 0.001). No significant difference was detected between local versus intravenous administration of umbilical cord–derived exosomes (*p* = 0.688).

**Table 1:** Endometrial epithelial thickness (µm) across study groups. Values are summarized per group (*n* = 5 animals/group). For each animal, endometrial epithelial thickness represents the mean of measurements obtained from 5 distinct microscopic fields.

Group	Mean ± SD (µm)	Median [Q1–Q3] (µm)	Min–Max (µm)
N-C	404,0 ± 40,3	391,1 [381,5–411,0]	366,4–470,1
P-C	135,6 ± 26,5	126,2 [123,6–138,2]	110,5–179,7
umb-msc	310,2 ± 15,5	305,6 [302,6–305,6]	299,6–337,6
umb-exo	320,1 ± 25,4	312,9 [302,5–322,8]	299,8–362,6
adipose-msc	336,3 ± 44,4	318,4 [314,7–320,9]	311,7–415,5
adipose-exo	343,0 ± 27,9	349,8 [340,4–361,4]	296,6–366,8
IV-umb-exo	339,3 ± 65,9	317,9 [316,4–321,1]	286,6–454,6

N-C, negative control; P-C, positive control (IUA only); umb-msc, local umbilical cord–derived MSC; umb-exo, local umbilical cord–derived exosome; adipose-msc, local adipose-derived MSC; adipse-exo, local adipose-derived exosome; iv-umb-exo, intravenous umbilical cord–derived exosome.

### Uterine wall thickness

Uterine wall thickness was calculated as the mean of five measurements obtained from different microscopic fields for each animal. Summary statistics are presented in Table 2. The positive control group showed a marked reduction in uterine wall thickness compared with the negative control group (*p* < 0.001). All treatment groups demonstrated significantly greater uterine wall thickness than the positive control group (all *p* < 0.006). No significant difference was observed between local and intravenous administration of umbilical cord-derived exosomes (*p* = 0.746).

**Table 2:** Uterine wall thickness (µm) across study groups. Values are summarized per group (*n* = 5 animals/group). For each animal, uterine wall thickness represents the mean of measurements obtained from 5 distinct microscopic fields.

Group	Mean ± SD (µm)	Median [Q1–Q3] (µm)	Min–Max (µm)
N-C	709.1 ± 14.2	702.6 [699.6–711.3]	699.2–732.9
P-C	253.8 ± 16.5	256.6 [243.0–266.6]	231.6–271.5
umb-msc	605.2 ± 4.6	604.6 [602.8–607.9]	599.2–611.3
umb-exo	628.1 ± 19.6	627.9 [623.1–636.9]	599.6–652.9
adipose-msc	632.9 ± 7.0	632.8 [629.5–636.9]	623.5–641.9
adipose-exo	680.8 ± 32.3	692.5 [690.8–694.6]	623.6–702.8
IV-umb-exo	633.2 ± 7.1	632.8 [632.7–635.9]	622.6–642.1

N-C, negative control; P-C, positive control (IUA only); umb-msc, local umbilical cord–derived MSC; umb-exo, local umbilical cord–derived exosome; adipose-msc, local adipose-derived MSC; adipose-exo, local adipose-derived exosome; iv-umb-exo, intravenous umbilical cord–derived exosome.

### Histochemical findings (H&E and Masson’s trichrome)

On H&E staining, the negative control group exhibited normal uterine histomorphology, whereas the positive control (IUA-only) group showed marked fibrosis involving the endometrium and other layers, low cuboidal epithelium with focal epithelial loss, reduced gland numbers, and luminal narrowing/occlusive changes, consistent with successful establishment of the intrauterine adhesion model. In the treatment groups, these abnormalities were attenuated to varying degrees, with restoration toward a higher columnar endometrial epithelium and increased glandular presence compared with the positive control group.

Masson’s trichrome staining demonstrated minimal collagen deposition in the negative control group and markedly increased collagen accumulation in the positive control group. In the treatment groups, collagen distribution was generally reduced compared with the positive control group, although the degree of reduction varied among groups. Representative H&E and Masson’s trichrome images are shown in Supplementary Fig. 1.

### Immunohistochemical findings (Collagen I, FGF-2, HIF-1α, VEGF)

Collagen type I immunoreactivity was markedly increased in the positive control group. Collagen distribution in the umbilical cord–MSC group was close to the negative control group. The umbilical cord–exosome and adipose-derived exosome groups showed broadly similar patterns. The adipose-derived MSC group demonstrated focal areas of intense Collagen I expression. The intravenous umbilical cord–exosome group showed collagen intensity lower than the positive control but higher than the negative control.

For FGF-2, HIF-1α, and VEGF, the highest expression was observed in the positive control group and the lowest in the negative control group. Among the treatment groups, staining intensity generally followed the same descending order: umbilical cord–MSC > umbilical cord–exosome > intravenous umbilical cord–exosome > adipose-derived MSC > adipose-derived exosome. (Collagen I, FGF-2, HIF-1α, VEGF images are presented in Supplementary Fig. 2).

### Semi-quantitative scoring: inflammation, vascular proliferation, and fibrosis

**Fig. 2:**
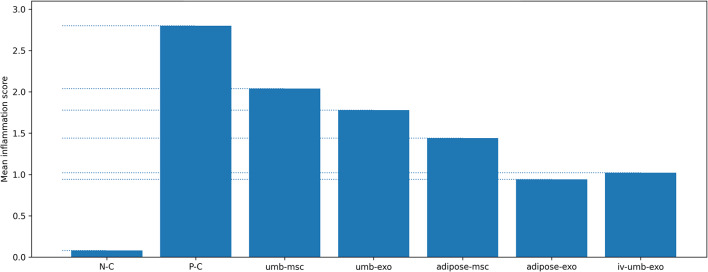
Inflammation score across experimental groups: Semi-quantitative inflammation scores (0–3; 0: none, 1: mild, 2: moderate, 3: marked) in uterine tissue sections. Scores were assigned based on microscopic evaluation of inflammatory cell infiltration and summarized at the group level (*n* = 5 rats/group). Bars represent group means.

For inflammation scoring, significant differences were present between the negative control, positive control, and treatment groups overall (*p* < 0.001). Among treatment arms, no significant differences were observed for umbilical cord–MSC vs. umbilical cord–exosome (*p* = 0.317), adipose-derived MSC vs. umbilical cord–exosome (*p* = 0.090), and adipose-derived exosome vs. intravenous umbilical cord–exosome (*p* = 0.993), whereas other pairwise comparisons were significant (*p* < 0.001). Based on the magnitude of reduction in inflammatory findings, the treatment groups ranked as: adipose-derived exosome > intravenous umbilical cord–exosome > adipose-derived MSC > umbilical cord–exosome > umbilical cord–MSC. (Fig. 2)

**Fig. 3:**
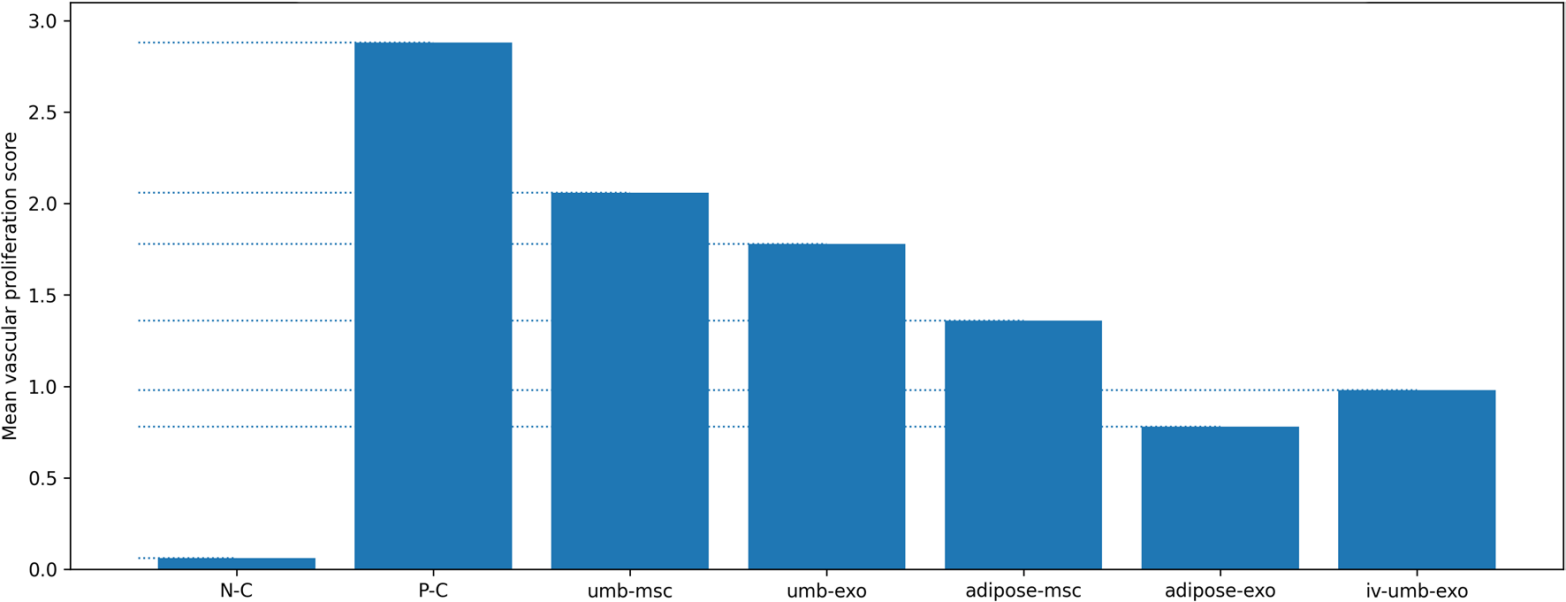
Vascular proliferation score across experimental groups: Semi-quantitative vascular proliferation scores (0–3; 0: none, 1: mild, 2: moderate, 3: marked) in uterine tissue sections. Scores were assigned based on microscopic evaluation of vascular proliferation and summarized at the group level (*n* = 5 rats/group). Bars represent group means.

For vascular proliferation, overall group differences were significant (*p* < 0.001). The only non-significant comparison among treatments was umbilical cord–MSC vs. umbilical cord–exosome (*p* = 0.124); other pairwise comparisons were significant (*p* < 0.001). Vascular proliferation increased from lowest to highest across treatments as: adipose-derived exosome < intravenous umbilical cord–exosome < adipose-derived MSC < umbilical cord–exosome < umbilical cord–MSC. (Fig. 3)

**Fig. 4:**
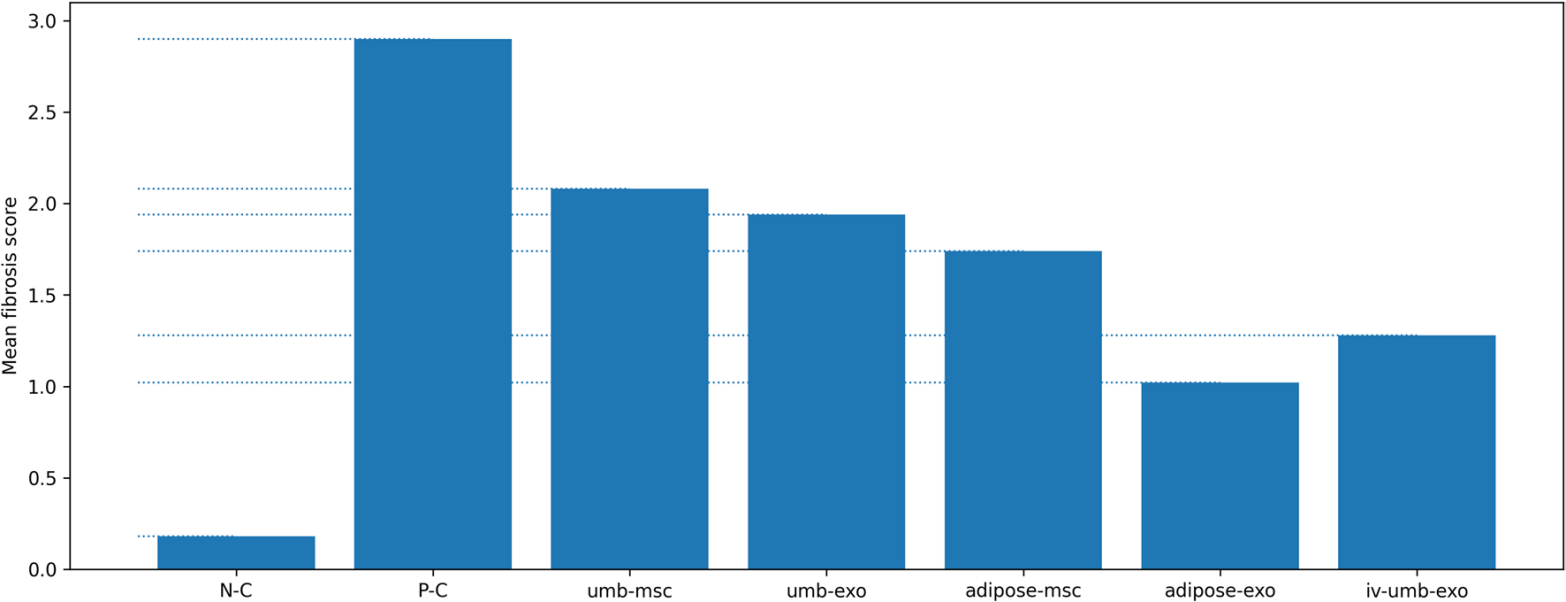
Fibrosis score across experimental groups: Semi-quantitative fibrosis scores (0–3; 0: none, 1: mild, 2: moderate, 3: marked) in uterine tissue sections. Scores were assigned based on microscopic evaluation of fibrotic changes and summarized at the group level (*n* = 5 rats/group). Bars represent group means.

For fibrosis scoring, overall differences between groups were significant (*p* < 0.001). Among treatment arms, umbilical cord–MSC vs. umbilical cord–exosome (*p* = 0.317), umbilical cord–exosome vs. adipose-derived MSC (*p* = 0.090), and adipose-derived exosome vs. intravenous umbilical cord–exosome (*p* = 0.993) were not significantly different; other pairwise comparisons were significant (*p* < 0.001). Fibrosis severity across treatments increased from lowest to highest as: adipose-derived exosome < intravenous umbilical cord–exosome < adipose-derived MSC < umbilical cord–exosome < umbilical cord–MSC. (Fig. 4)

## Discussion

Our data indicate that the therapeutic effect in this model is better captured by remodeling-oriented endpoints than by epithelial thickness alone. The clearest group separation was observed in fibrosis and inflammation scores, supported by collagen-sensitive histochemistry, whereas epithelial thickness was less discriminative across treatment arms. This suggests that a single administration may influence the stromal inflammatory-fibrotic response earlier and more strongly than surface re-epithelialization. In line with this, improved cavity organization and glandular architecture on H&E, together with reduced trichrome-positive collagen, point to decreased maladaptive extracellular matrix deposition as a key early effect.

To our knowledge, this study is among the first to compare adipose- and umbilical cord-derived MSC exosomes within the same IUA model and alongside their parent MSC therapies. Overall, exosome-based treatment showed a more favorable profile than MSC therapy across remodeling-related outcomes. Although the difference between adipose-Exo and UC-Exo was not statistically significant, adipose-Exo showed the most favorable numerical pattern for fibrosis and inflammation.

No direct head-to-head comparison of adipose- versus umbilical cord-derived MSC exosomes in IUA models was found in the provided references. Indirect evidence suggests that adipose-derived exosomes may support endometrial regeneration and fertility outcomes^1^, whereas umbilical cord-derived exosomes may be more consistently associated with anti-inflammatory and anti-fibrotic effects; however, these conclusions remain tentative in the absence of controlled comparative studies^13^.

MSC-derived extracellular vesicles (EVs) may support endometrial repair in intrauterine adhesions through several linked mechanisms^1,17,18^. Prior studies suggest that EVs can attenuate fibrotic remodeling by modulating TGF-β-related signaling and reducing extracellular matrix deposition, including collagen expression^17^. They may also mitigate inflammation by reducing pro-inflammatory cytokine signaling and promoting a repair-associated immune response^13,18,19^. In addition, EV cargo may enhance angiogenic and growth factor-related pathways that contribute to endometrial repair after injury^17,18,20^. This mechanistic framework is consistent with our findings, particularly the lower fibrosis and inflammation scores and the collagen-sensitive histochemical improvements observed in treated groups. However, the available evidence remains largely preclinical, and issues related to dosing, timing, and long-term safety require further study before clinical translation^17–20^. Recent experimental studies have likewise shown that regenerative or immune-modulating approaches may improve endometrial repair in rat models of Asherman syndrome/IUA by reducing fibrosis and inflammation while supporting angiogenesis and glandular restoration^21^.

The tissue source of MSCs can influence the content and biological behavior of their extracellular vesicles (EVs). Studies suggest that umbilical cord- and adipose-derived EVs carry different mixtures of miRNAs, growth factors, and immune-related signals^13,19^, which may contribute to distinct repair profiles in endometrial injury. Consistent with this, adipose-derived exosomes showed the most favorable numerical anti-fibrotic and anti-inflammatory pattern in our model, whereas cord-derived interventions also demonstrated robust repair-associated changes, including with systemic delivery. Prior studies have linked cord-derived EVs more strongly to immunomodulatory and pro-repair signaling, whereas adipose-derived EVs may exert relatively greater effects on angiogenesis and extracellular matrix turnover^1,18,20^. However, comparative evidence remains limited^1,18^, and these source-related differences should ultimately be interpreted together with functional reproductive outcomes in future studies.

Cell-based MSC therapy has been explored for endometrial repair, but many studies note limitations such as low cell survival and uncertain engraftment^1,18,19^. Increasingly, MSC benefit is thought to arise mainly through paracrine signaling rather than long-term tissue replacement^1,17,18^. In line with this, exosome-based treatment in our model produced tissue-level improvements broadly comparable to MSC-based treatment. This has supported interest in MSC-derived EVs/exosomes as a cell-free option that may be easier to store and may reduce some safety concerns linked to live-cell delivery^17,18,22^. Still, EV therapy has its own challenges, including variable isolation methods, unclear optimal dosing, and batch-to-batch differences^18,20^. Well-designed studies are needed to define standardized protocols and long-term safety^18,19^.

Delivery route may also influence treatment response. With systemic (IV) administration, vesicles can be diluted in circulation and cleared by other organs, potentially limiting uterine exposure^13,18^. Local intrauterine delivery may provide more direct contact and higher local concentration at the injury site^1,13,17^. In our study, route comparison was performed only within the umbilical cord-derived exosome groups, and the IV UC-exosome arm showed a remodeling profile broadly comparable to local administration. Some studies have also used gels, hydrogels, or scaffold-like carriers to improve retention and prolong tissue exposure^17,20^. Because our protocol involved a single administration without a retention system, further work is needed to optimize delivery for translation^18,22,23^.

Current literature on intrauterine adhesions still lacks direct head-to-head comparisons between whole mesenchymal stem cells and their derived extracellular vesicles, as well as controlled comparisons of tissue source and delivery route^1,17,18,22,23^. In this context, our comparative design helps clarify how these therapeutic variables may influence endometrial repair. However, future studies should prioritize long-term fertility outcomes and standardized dosing strategies to improve translational relevance and safety.

In our study, local versus intravenous delivery was compared only within the umbilical cord-derived exosome groups. No significant difference was observed in endometrial epithelial thickness (*p* = 0.688) or uterine wall thickness (*p* = 0.746). However, semi-quantitative remodeling scores showed a numerically more favorable pattern for inflammation and fibrosis in the IV UC-exosome group. Because no corresponding IV adipose-derived exosome group was included, these route-related findings should be interpreted cautiously and should not be generalized beyond the evaluated UC-exosome arm. Although local intrauterine administration may theoretically provide higher uterine exposure^1,18^, direct comparisons of local and systemic EV delivery remain limited^22,24^.

Several limitations should be considered when interpreting the present findings. First, the number of animals in each group was relatively small (*n* = 5), which may have reduced statistical power. Second, the study focused mainly on histological, immunohistochemical, and semi-quantitative tissue-level outcomes, whereas functional reproductive outcomes such as fertility, implantation, or pregnancy rates were not assessed. Although these findings provide important information regarding endometrial repair, they do not fully reflect restoration of reproductive function. Histological and histochemical findings were assessed using semi-quantitative scoring rather than digital image analysis, which may have limited objectivity despite blinded evaluation by two independent histologists. Third, the effects were evaluated after a single treatment administration and over a relatively short follow-up period, which may limit conclusions regarding the durability of the regenerative response. Moreover, although treatment volume was standardized across groups, detailed quantitative dose parameters such as MSC cell number and exosome concentration or particle number were not available, which may limit reproducibility. In addition, because not all treatment-source and administration-route combinations were represented, conclusions regarding route-related differences should be interpreted cautiously and should not be generalized beyond the groups studied. Finally, as with all preclinical animal models, the rat IUA model may not fully reproduce the complexity of human intrauterine adhesions. Further studies with larger sample sizes, longer follow-up, and functional reproductive endpoints are needed to confirm and extend these findings.

In conclusion, both MSC- and exosome-based treatments showed beneficial effects on endometrial repair in this experimental IUA model. Among the evaluated treatment groups, exosome-based approaches demonstrated a generally more favorable histological remodeling profile. However, these findings should be interpreted cautiously because of the small sample size, the absence of functional reproductive outcomes, and the limited route comparison. Further studies with larger sample sizes, longer follow-up, standardized characterization and dosing protocols, and reproductive outcome measures are needed to confirm these findings.

## Supplementary Information

Below is the link to the electronic supplementary material.

Supplementary Material 1

Supplementary Material 2

Supplementary Material 3

## Data Availability

The datasets generated and/or analyzed during the current study are available from the corresponding author on reasonable request.
