# Supplementary figures and images for: Endometrial regeneration with mesenchymal stem cells and exosomes: an experimental rat model of intrauterine adhesions
Source: Sci Rep. 2026 Mar 26;16:15016. doi: 10.1038/s41598-026-45939-7 (PMC13172503; doi:10.1038/s41598-026-45939-7)

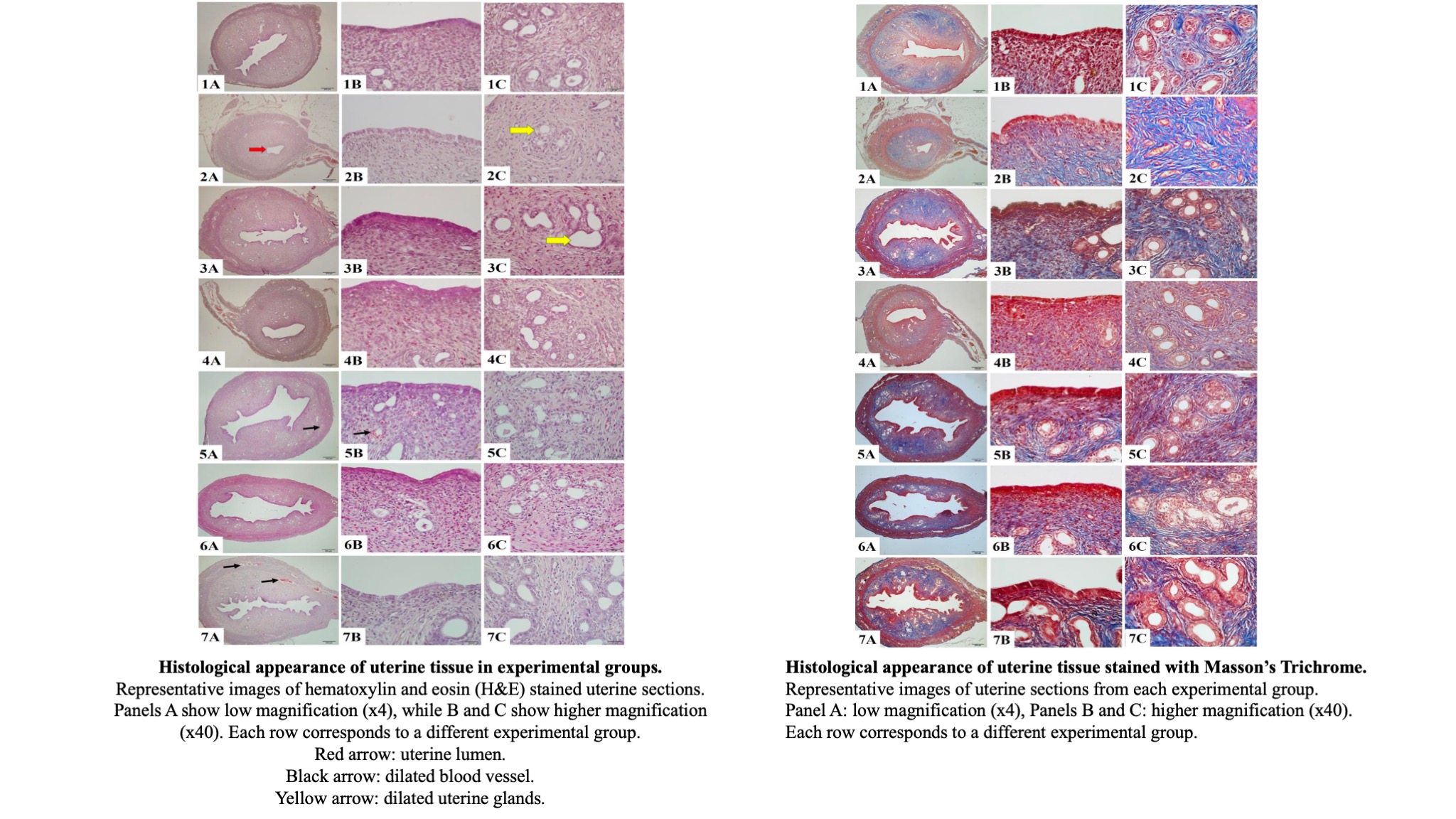

Supplement: Supplementary file 1 — Supplementary Material 1 [file 41598_2026_45939_MOESM1_ESM.jpg]

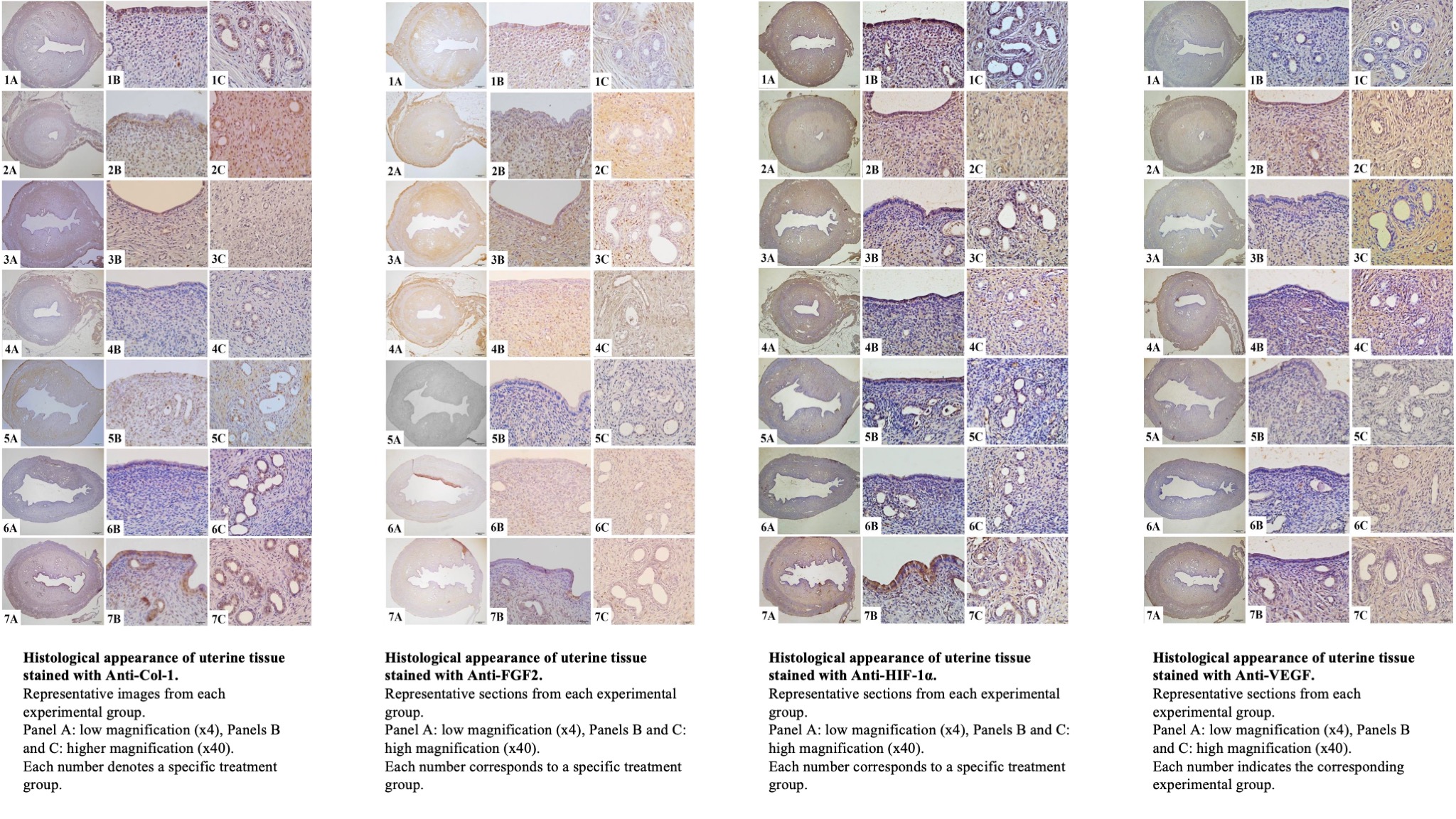

Supplement: Supplementary file 2 — Supplementary Material 2 [file 41598_2026_45939_MOESM2_ESM.jpg]
