# Supplementary material for: Endometrial regeneration with mesenchymal stem cells and exosomes: an experimental rat model of intrauterine adhesions
Source: Sci Rep. 2026 Mar 26;16:15016. doi: 10.1038/s41598-026-45939-7 (PMC13172503; doi:10.1038/s41598-026-45939-7)
Supplement: Supplementary file 3 — Supplementary Material 3 [file 41598_2026_45939_MOESM3_ESM.docx]

**Appendix A.Supplementary Methods**

S1. Animals and Housing Conditions

All experimental procedures were conducted at the Ege University Laboratory Animal Application and Research Center. Female Sprague–Dawley rats (approximately 12 weeks old, ~200 g) were housed in groups of five per cage under controlled environmental conditions (12-h light/12-h dark cycle, temperature 22 ± 2 °C) with ad libitum access to standard chow and water.

S2. Surgical Procedure and Induction of Intrauterine Adhesions

Following intraperitoneal anesthesia with ketamine hydrochloride (50 mg/kg) and xylazine hydrochloride (5 mg/kg), abdominal skin preparation was performed using povidone–iodine solution. A ~2 cm midline longitudinal laparotomy was carried out, and the uterus was exposed. No congenital or acquired genital tract anomalies were observed.

A 26G intravenous cannula was inserted into the intrauterine cavity near the tubal end. 0.3 mL of 95% ethanol was slowly administered under gentle pressure to induce chemical endometrial injury. The cannula was left in place for 3 minutes to allow adequate tissue exposure, while surrounding tissues were protected with sterile gauze to prevent intra-abdominal leakage. Subsequently, 0.5 mL of normal saline was infused through the same cannula to irrigate the uterine cavity.

The abdominal wall was closed anatomically using 4-0 vicryl sutures. For surgical site infection prophylaxis, all operated animals received penicillin 1000 U/kg intramuscularly. Animals were allowed to recover, and a 2-week interval was provided for adhesion formation.

S3. Therapeutic Administration Protocol

Two weeks after adhesion induction, a second laparotomy was performed in the relevant treatment groups using the same anesthesia and sterile technique. A cannula was placed into the intrauterine cavity, and prepared mesenchymal stem cell (MSC) or exosome suspensions were administered locally in a standardized volume of 0.2 mL according to group allocation.

In the systemic treatment group, umbilical cord MSC–derived exosomes were administered intravenously via the tail vein. Following each surgical procedure, the abdomen was closed with 4-0 vicryl sutures, and intramuscular penicillin (1000 U/kg) was administered.

S4. Isolation and Culture of Adipose-Derived MSCs

Adipose-derived MSCs were obtained from liposuction material generated in a concurrently conducted study in the same laboratory, following protocol amendment approval. Briefly, adipose tissue was washed repeatedly with phosphate-buffered saline (PBS) until the infranatant cleared. Tissue was then enzymatically digested with 0.075 mg/mL collagenase type I at 37 °C for 60 minutes with intermittent mixing.

The stromal vascular fraction was collected, enzyme activity was inhibited using α-MEM containing 20% fetal bovine serum (FBS), and cells were centrifuged and washed. Cell pellets were resuspended in α-MEM supplemented with 10% FBS, penicillin/streptomycin, and L-glutamine and cultured at 37 °C in a humidified atmosphere with 5% CO₂. Non-adherent cells were removed by media changes every other day until cultures reached approximately 90% confluence. Cells were detached using trypsin, and viability was assessed using trypan blue exclusion (>90% viability).

S5. Umbilical Cord–Derived MSC Culture

Human umbilical cord (Wharton’s jelly)–derived MSCs were supplied by Genkök Stem Cell Center. Cells were cultured at 37 °C under 5% CO₂ and high-humidity conditions, with media changes every 48 hours. Upon reaching 70–80% confluence, cells were passaged using 0.05% trypsin. Cell viability was evaluated using trypan blue staining prior to experimental use.

S6. Flow Cytometric Characterization of MSCs

Early-passage MSCs were characterized by flow cytometry to confirm mesenchymal phenotype. Cells were trypsinized, washed, and resuspended to a concentration of 1 × 10⁶ cells per test. Samples were incubated with specific monoclonal antibodies against CD44, CD73, CD90 and CD105, along with appropriate isotype controls. PE-only staining was negligible, indicating low background signal. Following fixation and washing steps, samples were analyzed using a flow cytometer, and data were interpreted using standard analysis software.

S7. Exosome Isolation

Exosomes were isolated from MSC-conditioned media using the miRCURY Exosome Cell Kit according to the manufacturer’s instructions. Briefly, conditioned media were centrifuged at 3000g for 10 minutes to remove cellular debris. The supernatant was mixed with precipitation buffer, incubated overnight at +4 °C, and centrifuged at 3200g for 30 minutes. The resulting exosome-containing pellet was resuspended in 100 µL of resuspension buffer. Exosome stocks were stored at −20 °C.

S8. Exosome Characterization

Exosome morphology and spherical structure were evaluated using scanning electron microscopy (SEM; Thermo Scientific Apreo S). Samples intended for characterization were stored at +4 °C until analysis. In addition, exosome-associated surface markers were assessed by flow cytometry using antibodies against CD9 and CD63 (Beckman Coulter). For flow cytometric analysis, exosome suspensions were incubated with fluorochrome-conjugated anti-CD9 and anti-CD63 antibodies for 30 minutes at room temperature in the dark. Within the gated population, CD9 positivity was 71.18% and CD63 positivity was 79.14%.

S9. Histological Processing and Staining

After sacrifice, uterine tissues were fixed in 4% paraformaldehyde for 24–48 hours and processed using routine dehydration, clearing, and paraffin embedding protocols. Sections (5 µm thickness) were obtained using a microtome and mounted on poly-L-lysine–coated slides.

Hematoxylin–eosin staining was used to assess general tissue morphology, while Masson’s trichrome staining was performed to evaluate collagen deposition and fibrosis.

S10. Immunohistochemistry

Immunohistochemical staining was performed using primary antibodies against collagen type I, FGF-2, VEGF, and HIF-1α following antigen retrieval, endogenous peroxidase blocking, and serum blocking steps. Detection was achieved using a streptavidin–biotin–peroxidase system with DAB as the chromogen. Sections were counterstained with hematoxylin.

S11. Semi-Quantitative Histological Scoring

Inflammation, vascular proliferation, and fibrosis were evaluated using predefined semi-quantitative scoring systems (0–3). Ten randomly selected fields per specimen were analyzed. Immunohistochemical staining intensity and distribution were assessed in five randomly selected fields per slide at ×40 magnification. All histological evaluations were performed by two independent histologists blinded to group allocation.

S12. Statistical Analysis

Detailed statistical procedures, including normality testing, variance homogeneity assessment, parametric and non-parametric comparisons, post hoc analyses, and chi-square testing for categorical score distributions, were performed as described in the main manuscript.
